# c-Abl Deficiency Provides Synaptic Resiliency Against Aβ-Oligomers

**DOI:** 10.3389/fncel.2019.00526

**Published:** 2019-11-26

**Authors:** Daniela A. Gutierrez, Lina M. Vargas, América Chandia-Cristi, Catalina de la Fuente, Nancy Leal, Alejandra R. Alvarez

**Affiliations:** Cell Signaling Laboratory, Faculty of Biological Science, Department of Cell and Molecular Biology, Center for Aging and Regeneration (CARE), Pontificia Universidad Católica de Chile, Santiago, Chile

**Keywords:** Alzheimer’s disease, c-Abl tyrosine kinase, synapse, Aβ-oligomers, dendritic spines

## Abstract

Spine pathology has been implicated in the early onset of Alzheimer’s disease (AD), where Aβ-Oligomers (AβOs) cause synaptic dysfunction and loss. Previously, we described that pharmacological inhibition of c-Abl prevents AβOs-induced synaptic alterations. Hence, this kinase seems to be a key element in AD progression. Here, we studied the role of c-Abl on dendritic spine morphological changes induced by AβOs using c-Abl null neurons (c-Abl-KO). First, we characterized the effect of c-Abl deficiency on dendritic spine density and found that its absence increases dendritic spine density. While AβOs-treatment reduces the spine number in both wild-type (WT) and c-Abl-KO neurons, AβOs-driven spine density loss was not affected by c-Abl. We then characterized AβOs-induced morphological changes in dendritic spines of c-Abl-KO neurons. AβOs induced a decrease in the number of mushroom spines in c-Abl-KO neurons while preserving the populations of immature stubby, thin, and filopodia spines. Furthermore, synaptic contacts evaluated by PSD95/Piccolo clustering and cell viability were preserved in AβOs-exposed c-Abl-KO neurons. In conclusion, our results indicate that in the presence of AβOs c-Abl participates in synaptic contact removal, increasing susceptibility to AβOs damage. Its deficiency increases the immature spine population reducing AβOs-induced synapse elimination. Therefore, c-Abl signaling could be a relevant actor in the early stages of AD.

## Introduction

In the adult central nervous system, synaptic connections are highly dynamic, allowing the brain to reorganize and integrate new information. The formation, stabilization, weakening and elimination of synapses play a key role in neuronal transmission and wiring (Fernandes and Carvalho, 2016). Excitatory synaptic contacts have actin-enriched structures known as dendritic spines, which are constantly modified. Dendritic spines arise from the dendritic shaft and have immature forms known as filopodia and thin spines. The mature forms known as mushroom spines have post-synaptic densities enriched in scaffolding proteins and glutamate receptors (Harris, 1999; Hayashi and Majwska, 2005). The formation, maturation, shape-changing and pruning of dendritic spines have been associated with learning and memory (Riccomagno and Kolodkin, 2015; Piochon et al., 2016). Interestingly, processes that alter the size, shape and density of dendritic spines such as the remodeling of synaptic complexes, and changes in actin cytoskeleton, have been implicated in synaptic plasticity, synaptic dysfunction and neuronal death (Kommaddi et al., 2018). In fact, synaptic dysfunction has been associated with the genesis and progression of different neurodegenerative diseases (Hardy and Selkoe, 2002; Clare et al., 2010; Henstridge et al., 2018).

Alzheimer’s disease (AD) is characterized by synaptic and neuronal loss in brain regions related with memory and cognition (Gomez-Isla et al., 2008; Viola and Klein, 2015). Cognitive decline strongly correlates with the loss of pre- and post-synaptic markers in AD brains (Sze et al., 1997; Masliah et al., 2001; Reddy et al., 2005; Calabrese et al., 2007). And it also correlates with early changes in glutamatergic function (Kirvell et al., 2006). Neuronal cultures are mostly excitatory, and Aβ_1–42_ oligomers (AβOs) bind mainly to synapses that use glutamate as a neurotransmitter (Lacor et al., 2007). Synaptic binding of AβOs in hippocampal neurons decreases surface N-Methyl-D-aspartate (NMDA) receptors and EphB2 protein levels (Calabrese et al., 2007; Lacor et al., 2007); it also reduces levels of PSD95 and the GluR1 subunit of the AMPA receptor (Almeida et al., 2005). Furthermore, there is dendritic spine loss in the brains of AD patients and in mice models of AD that express the mutant human Amyloid Precursor Protein (APP; Davies et al., 1987; Moolman et al., 2004; Jacobsen et al., 2006). Neuronal degeneration begins with early neuronal dysfunction mediated by AβOs, which disrupts the synapse and inhibits long-term potentiation (LTP; Lambert et al., 1998; Selkoe, 2008; Ferreira and Klein, 2011; Tu et al., 2014). AβOs bind to dendritic spines, significantly decreasing the number of synaptic terminals, reducing dendritic spine density in neurons and changing their morphology to more elongated shapes (filopodia spines; Spires et al., 2005; Jacobsen et al., 2006; Lacor et al., 2007). Altogether, these data suggest that early synaptic dysfunction and synaptic loss induced by AβOs are involved in the cognitive decline in AD patients.

Previously, we identified c-Abl as a key signaling molecule involved in AD pathology (Alvarez et al., 2004; Cancino et al., 2008; Vargas et al., 2014; Gonzalez-Zuñiga et al., 2014). The ABL family of non-receptor tyrosine kinases, includes c-Abl and the Abl-related gene (Arg). Deletion of c-Abl or Arg in mice causes hematopoietic or behavioral defects, respectively (Tybulewicz et al., 1991). The complete deletion of both kinases causes lethal neurulation defects due to actin accumulation in the neuroepithelium and defective closure of the neural tube. Therefore, animals die from hemorrhaging at embryonic day E11 (Koleske et al., 1998). On the other hand, the knockout conditional mice model (c-Abl-KO) used here, is under the promoter of neuronal and glial progenitor cells Nestin-CRE; and does not present neurulation defects or embryonic lethality (Qiu et al., 2010).

c-Abl is a key signal transducer for growth factors, adhesion and axon-guidance receptors as well as for DNA damage, oxidative stress and others (Wang, 2014). Since it has G- and F-actin binding domains, c-Abl can interact with cytoskeletal related proteins. It phosphorylates Abi and WAVE2 and therefore activates Arp2/3 to regulate actin polymerization (Mendoza, 2013). c-Abl is expressed at high levels in neurons, especially in pre- and post-synaptic terminals and its activity inactivates RhoA promoting dendrogenesis and synaptic plasticity (Moresco and Koleske, 2003; Jones et al., 2004; Lin et al., 2013). Furthermore, unlike Arg, c-Abl has the ability to shuttle between the cytoplasm and the nucleus regulating chromatin (Tip60), gene expression and apoptosis (p73/p53; Jiang et al., 2011; Wang, 2014).

Interestingly, the c-Abl activation pathway has also been involved in neuronal apoptosis linked to the pathology of different neurodegenerative diseases (Schlatterer et al., 2011). In the case of AD, we described that Aβ fibrils-induced c-Abl-activation triggers apoptosis in neuronal cultures (Alvarez et al., 2004; Jing et al., 2009). Also, c-Abl stabilizes HDAC2 repressing neuronal gene expression, contributing to memory impairment in AD (Gonzalez-Zuñiga et al., 2014). We have also demonstrated that c-Abl plays a key role in synaptic structural changes induced by AβOs. In this pathway, AβOs bind to the EphA4 receptor causing downstream activation of c-Abl, synaptic loss and LTP blockade (Vargas et al., 2014). Moreover, EphA4/c-Abl signaling is inherently activated in APPswe/PSEN1ΔE9 transgenic mice, and thus, c-Abl inhibition resulted in decreased AβOs accumulation in the brain (Cancino et al., 2008; Fu et al., 2014; Yáñez et al., 2016). On the other hand, c-Abl inhibition by Imatinib (ATP binding site c-Abl inhibitor), improves behavioral impairments in Aβ-fibrils injected animals, and also reduces AβOs-mediated spine density loss (Cancino et al., 2008). However, Imatinib does not only inhibits c-Abl, but also Arg, c-kit, PDGFR and Src (Greuber et al., 2013; Lin and Roux, 2013).

Here, we show that c-Abl is present at synapses and co-localizes with the post-synaptic protein PSD95. To further dissect the role of c-Abl on synaptic changes induced by AβOs, we used c-Abl null hippocampal neurons. In the absence of c-Abl, these neurons showed increased spine density and enrichment of immature spines. Even though AβOs disrupt the synapse, c-Abl null neurons remodel dendritic spines, decreasing the number of mature spines and increasing the number of immature spines; but maintaining their synaptic contacts as a way to overcome the synaptic damage induced by AβOs.

## Materials and Methods

### Animals

c-Abl null mice were bred from c-Abl^loxp^/c-Abl^loxp^ and Nestin-Cre^+^ mice, kindly donated by Dr. AJ Koleske (Yale School of Medicine, New Haven, CT, USA). Genotyping was performed using a polymerase chain reaction (PCR)-based screening (Bradley and Koleske, 2009). Primers: Abl1-floxA: 5′-GATGTCTCTACAGGGTTTAAGATTAAGAGCA-3′; and Abl1-floxB: 5′-AGTTAACACACCTCCAGAGTGAGTGCCCT-3′; Cre: B: 5′-GCAATTTCGGCTATACGTAACAGGG-3′; and A: 5′-GCAAGAACCTGATGGACATGTTCAG-3′.

All protocols were approved and followed local guidance documents generated by the *ad hoc* Chilean committee (CONICYT), and were approved by the Bioethics and Care of Laboratory Animals Committee of the Pontificia Universidad Católica de Chile (Protocol #150721002). We followed the recommendations of the Guide for Care and Use of Laboratory Animals from US Public Health Service.

### Primary Hippocampal Cell Culture

Hippocampi from c-Abl knockout (c-Abl^loxp^/c-Abl^loxp^; Nestin-Cre^+^; c-Abl-KO) and their WT siblings (c-Abl^floxo/floxo^; WT) mice embryos at day 18 (E18) were dissected, and primary hippocampal cultures were prepared as previously described (Kaech and Banker, 2006). This conditional c-Abl-KO mice model does not present the c-Abl protein in the brain, unlike their WT littermates, although it is present in other tissues (see Supplementary Figure S1). Pregnant mice were anesthetized with CO_2_ and subsequently euthanized by cervical dislocation. Cultures were maintained at 37°C in 5% CO_2_ with neurobasal growth medium (Invitrogen, Carlsbad, CA, USA), supplemented with B27, 2 mM L-glutamine, 100 U/ml penicillin, and 100 μg/ml streptomycin (Invitrogen, Carlsbad, CA, USA). On the next day, cultured neurons were treated with 1 μM AraC to prevent glial cell proliferation. Hippocampal neurons were treated with AβOs at 5 μM final concentration for 5 h.

### Aβ Oligomers Preparation

Human synthetic Aβ_1–42_ peptide (Genemed Biotechnologies Inc, San Francisco, CA, USA) was suspended in 1, 1, 1, 3, 3, 3 hexafluoro-2-propanol 0.5 mg/ml (Sigma-Aldrich, St. Louis, MO, USA). Peptide samples were vortexed to obtain a homogenous solution, aliquoted into microfuge tubes and lyophilized. The Aβ_1–42_ peptide aliquots were resuspended in nanopure water at 200 μM concentration and vortexed briefly. Aggregation was allowed to proceed for 12 h at 4°C following the protocols by Arimon et al. (2005) and Sokolov et al. (2006). To form fluorescent AβOs (AβOs-FITC), synthetic Aβ_1–42_ coupled to FITC (Bachem, Torrance, CA, USA) was used. Gel electrophoresis was performed at 4°C in Tris-tricine gels (4% stacking, 10% spacer and 16% resolutive gel) at 50 V to 80 V. Aβ_1–42_ species were transferred onto nitrocellulose membrane (0.22 μm pore) for 1 h and 350 mA. Blocking was performed in TBS-3% non-fat milk, and incubated with the primary antibody WO2 raised against Amyloid-β-peptide (MABN10, Millipore, Burlington, MA, USA 1:1,000; Supplementary Figure S2).

### Neuronal Dendritic Spine Labeling and Quantification

Hippocampal neurons from WT and c-Abl-KO embryos (E18) were seeded onto poly-L-lysine-coated coverslips in 24-well culture plates at a density of 10^4^ cells per well. For transfection of pcDNA 3.0 GFP-plasmids, we used the Magnetofection^TM^ technology with the reagent Neuromag (OZ Biosciences, NM50200) in 18 DIV neurons. After 24 h, these neurons were treated with 5 μM AβOs for 5 h. For dendritic spine quantification, we analyzed GFP-expressing neurons and the co-localization with PSD95 or TRITC-phalloidin (ECM Biosciences, Versailles, KY, USA) to label actin cytoskeleton and examine spine morphology. Coverslips were mounted with mounting medium (DAKO) and then observed using an Olympus IX81 LSM Fluoview or a Nikon Eclipse C2 Ti-E confocal microscope. Images were processed with ImageJ (NIH). Antibodies used for immunofluorescence were anti-Piccolo [epitope 44aII antibody (Cases-Langhoff et al., 1996; Gundelfinger et al., 2016) produced by Viviana I. Torres and Craig C. Garner]; anti-PSD95 (75–028) from NeuroMab, Davis, CA, USA. Dendrite and spine morphology classification was performed according to the method described by Tyler and Pozzo-Miller (2003).

### Immunoblot Analysis

Hippocampal neurons from WT and c-Abl-KO embryos were plated at a density of 10^6^ cells/cm^2^. At different DIV, they were washed and lysed in radioimmunoprecipitation assay (RIPA) buffer (50 mM Tris, 150 mM NaCl, 1 mM EGTA, 1 mM EDTA, 0.5% deoxycholate, 1% NP-40, and 0.1% SDS) supplemented with protease inhibitors. Cell lysates were centrifuged at 14,000 rpm for 15 min at 4°C. Protein quantification was performed using the Pierce^®^ BCA Protein Assay Kit (Thermo Fisher Scientific, Waltham, MA, USA). The fractions were subjected to SDS-PAGE and transferred onto nitrocellulose membranes (Thermo Fisher Scientific, Waltham, MA, USA). The antibodies used were: anti-βIII-tubulin (AA10 sc80016, Santa Cruz Biotechnology, Dallas, TX, USA), anti-c-Abl (A5844, Sigma-Aldrich, St. Louis, MO, USA); anti-PSD95 (75–028) and anti-SAP102 (75–058), from NeuroMAb Antibodies Inc.

### Apoptotic Nuclei Quantification

Hippocampal neurons from WT and c-Abl-KO embryos were seeded onto poly-L-lysine-coated coverslips in 24-well culture plates at a density of 5 × 10^4^ cells per well and treated with 5 μM AβOs for 5 h. Cells were fixated and immunostained with active caspase-3 (AB3623, Millipore, Burlington, MA, USA) and Hoechst (33342, Thermo Fisher Scientific, Waltham, MA, USA), to visualize apoptotic nuclei.

### RT-PCR

Total RNA from 10 DIV WT and c-Abl-KO hippocampal neurons was extracted using TRIzol (Life Technologies, Carlsbad, CA, USA, 15596). Neurons were treated with 5 μM AβOs for 1, 3, 5 and 12 h. Fibrillary forms of the Aβ-peptide (Aβf) were used as control at 5 μM. Total RNA was reverse-transcribed into cDNA using the High Capacity cDNA Reverse Transcription kit (Applied Biosystems). Primers: c-Abl Forward: 5′ AGCATCACTAAAGGCGAGAA 3′; c-Abl Reverse: 5′ CACCCTCCCTTCATACCG 3′; GAPDH Forward: 5′ GGGTGTGAACCACGAGAAATA 3′; GAPDH Reverse: 5′ CTGTGGTCATGAGCCCTTC 3′.

### Statistical Analyses

We use ImageJ for dendritic spine quantification every 10 μm of each secondary dendrite. We performed three independent experiments (mice cultures) with a total of four to five embryos per condition, and two to five dendrites per neuron analyzed for a total of 13–17 neurons per condition. The total number of spines is indicated in each figure. For spine profile analyses, we used quantifications for each type (Mushroom, Stubby, Thin, Filopodium and Branched) and normalized with the total number of dendritic spines per dendrite analyzed per neuron. All data are presented as Mean ± Mean Standard Error (SEM). Mean, SEM and SE values and the number of experiments are indicated in each figure. Spine quantification statistical analyses were performed using Mann–Whitney unpaired *t*-test for dendritic spine type analyses and two-way ANOVA, followed by Tukey’s *post hoc* multiple comparison test using GraphPad Prism 6 software for genotype/treatment comparisons. Pearson’s and Mander’s colocalization were performed using the ImageJ plugin COLOC2. The significance level was *P* < 0.05 for all treatments.

## Results

### c-Abl Is Located in Neurites and Its Absence Increases Spine Density

**Figure 1:**
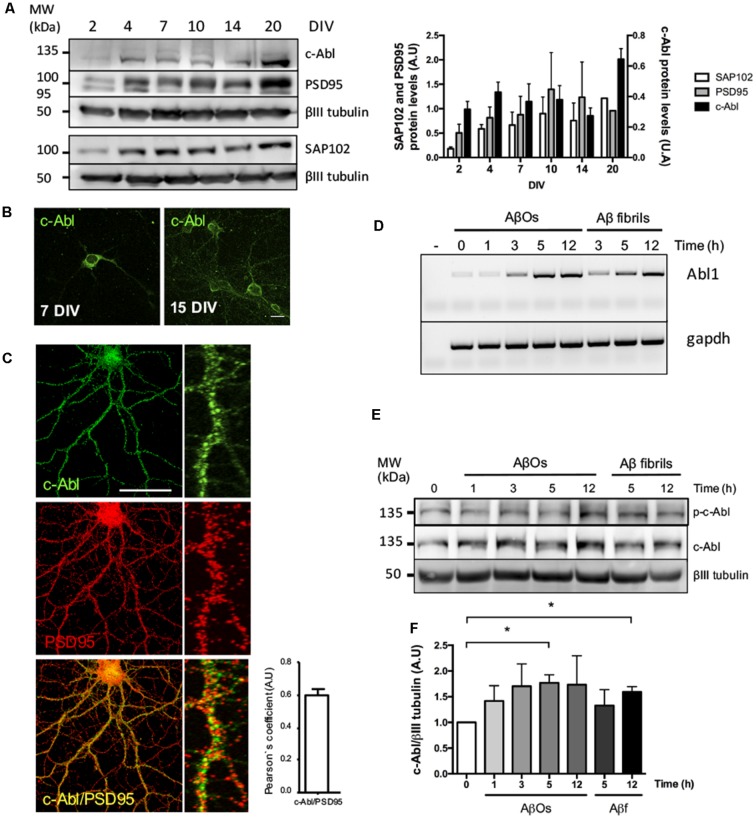
c-Abl is located at the synapse and its mRNA and protein levels increase after Aβ oligomers (AβOs) treatment. **(A)** c-Abl, PSD95 and SAP102 protein levels increase as the neuronal culture ages (from 7 to 20 DIV). The graph shows quantification of protein levels. *n* = 3 **(B)** Immunofluorescence of hippocampal neurons showing c-Abl (in green) from soma to dendrites at 15 DIV. **(C)** Immunofluorescence showing c-Abl (in green) and PSD95 (in red) in hippocampal neurons at 19 DIV. The graph shows protein colocalization by Pearson’s correlation. Scale bar = 20 μm and 2 μm for magnifications. **(D)** Hippocampal cultured neurons were treated with 5 μM AβOs at indicated times. mRNA expression for *Abl1* gene was assayed by RT-PCR using *gapdh* as loading control (water negative control). **(E)** Western-blot of c-Al total protein levels normalized to βIII tubulin, as the graph shows **(F)**. Neurons incubated with 5 μM fibrillary forms of the Aβ-peptide (Aβf) were used as control. Unpaired *t*-test, *p < 0.5.

First, in order to examine the role of c-Abl in the synapse, we analyzed its protein levels over time. During culture maturation, the post-synaptic scaffold protein SAP102 and the post-synaptic density protein-95 (PSD95), continuously increase their protein levels. Meanwhile, c-Abl displays the highest protein levels during neurite extension at 4 DIV and at full culture maturation at 20 DIV (Figure 1A). At 7 DIV c-Abl is found mainly located at the soma, while at 15 DIV c-Abl is broadly distributed, not only in the soma, but it also has a punctate shape in all neuronal processes (Figure 1B). As shown by Pearson’s correlation (Figure 1C), c-Abl localizes in the post-synaptic compartment, where it co-localizes with PSD95.

**Figure 2:**
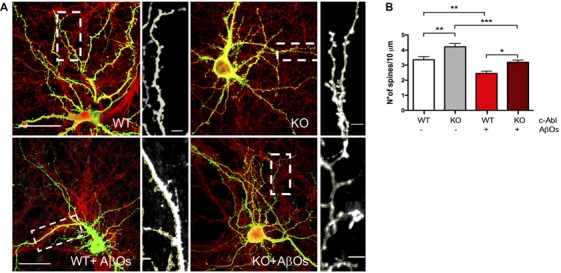
Aβ oligomer-induced dendritic spine density decrease is independent of c-Abl. **(A)** GFP-transfected wild-type (WT) and c-Abl-KO neurons were treated with Aβ oligomers (AβOs) for 5 h, and dendritic spines were counted (PSD95 is shown in red). The sections of secondary dendrites (rectangle) were delimited to show dendritic spine morphology. Complete image scale bar = 20 μm, magnifications scale bar = 2 μm. **(B)** Quantification of spine density (number of spines/10 μm dendrite) shows that c-Abl-KO neurons show higher spine density (4.22 ± 0.22 spines/10 mm dendrite) than WT neurons (3.36 ± 0.20 spines/10 mm dendrite). On the other hand, AβOs treatment significantly reduces spine density in both, WT and c-Abl-KO neurons (2.45 ± 0.15 and 3.19 ± 0.15 spines/10 mm, respectively; *n* = 47 WT; *n* = 49 WT+AβOs; *n* = 49 KO, and *n* = 53 dendrites for KO+AβOs). Two-way ANOVA and Tukey’s multiple comparisons. *p < 0.5; **p < 0.01; ***p < 0.001. *n* = 3 independent cultures, 4–5 mice embryos per condition.

To investigate whether c-Abl could be regulating dendritic spine density, we studied spine morphology in cultured c-Abl knock-out (c-Abl-KO) hippocampal neurons transfected with GFP expression plasmids at 18 DIV to label whole single neurons (Figure 2A). One day later, we counted their dendritic spine population in secondary branches. Interestingly, we found that c-Abl deficiency increases dendritic spine density. c-Abl-KO neurons showed an enriched spine density with 4.22 ± 0.25 spines/10 μm dendrite (Figure 2B), in comparison with WT neurons that showed 3.36 ± 0.20 spines/10 μm dendrite. Therefore, c-Abl-KO neurons display a 25.6% dendritic spine increase in their spine density (Figure 2B).

### Dendritic Spine Loss Induced by AβOs Is Independent of c-Abl

Exposure to AβOs significantly decreases dendritic spine density in neurons. Using a synthetic Aβ_1–42_ human peptide (see “Materials and Methods” section), we prepared an overnight solution of AβOs characterized by the presence of dimers and trimers (Supplementary Figure S2). These soluble species of the peptide have been previously described as the most toxic species of AβOs that bind to the synapse of hippocampal neurons (Tu et al., 2014; Mi et al., 2017). Previously, we described that AβOs induce c-Abl activation participating in the loss of synapses (Vargas et al., 2014). Moreover, the binding of FITC-labeled AβOs to dendrites activates c-Abl (p-c-Abl; Figure 1E, Supplementary Figure S3). Thus, we treated hippocampal neurons with AβOs and observed a quick increase (at 1 h) in c-Abl phosphorylation that remains active 5 h after treatment with AβOs. This increase in active c-Abl was also associated with a later increase in both Abl1 mRNA and c-Abl protein levels from 3 h post-AβOs incubation (Figures 1D–F). Fibrillary forms of the Aβ-peptide (Aβf) also increased c-Abl protein levels by 3 h incubation and were used as a control.

Then, we asked whether c-Abl ablation modulates AβOs-induced synapsis loss. GFP-expressing WT and c-Abl-KO hippocampal neurons were exposed to AβOs and after 5 h treatment, the number of dendritic spines was evaluated. Primary dendrites were the least affected by the AβOs treatment, while tertiary and far-away branches were the most affected, and even disrupted by AβOs. Therefore, we quantified dendritic spines in secondary branches. WT neurons showed reduced spine density after AβOs treatment (3.36 ± 0.20 vs. 2.45 ± 0.15 spines/10 μm dendrite), representing 27% spine loss. As well as WT neurons, c-Abl-KO neurons displayed a significant reduction over spine density (4.22 ± 0.22 vs. 3.19 ± 0.15 spines/10 μm dendrite), representing a 24.3% spine loss (Figure 2B, Supplementary Figure S4C). Thus, AβOs treatment induced a decrease in the number of dendritic spines in both, WT and c-Abl-KO neurons. Since we found only a 3% difference in dendritic spine density between c-Abl-KO and WT neurons treated with AβOs, suggesting that AβOs-driven spine loss seems to be independent of c-Abl.

### AβOs-Induced Immature Spines Are Influenced by the Presence of c-Abl

Then, we evaluated spine morphology by analyzing the dendritic spines shape profile following five major categories: mushroom, stubby, branched, thin and filopodia spines. Mature spines are mushroom, big spines in which the head is wider than the neck. Intermediate states are stubby, smaller and neckless spines. Branched spines are two-headed spines. Finally, immature spines are thin protrusions shorter than 2 μm length; and filopodium, in which protrusions are longer than 2 μm length (Tyler and Pozzo-Miller, 2003).

**Figure 3:**
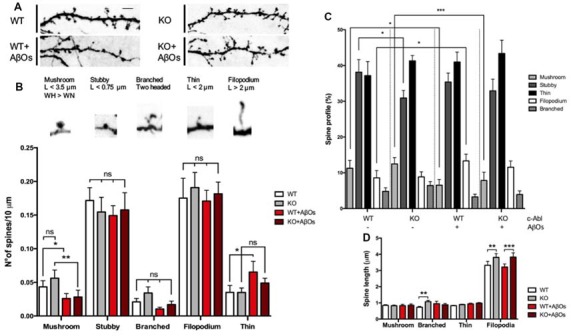
c-Abl modulates dendritic spine morphology: c-Abl deficiency increased formation of immature spines after Aβ oligomers treatment. **(A)** Dendrite sections of WT and c-Abl-KO neurons treated with AβOs present morphological variations on dendritic spines. Scale bar = 2 μm. Dendritic spines were classified into five categories: mushroom when the head (H) is wider (W) than the width of the neck (WN); stubby for neckless protrusions less than 0.75 μm length (L); thin for protrusions shorter than 2 μm; filopodium for protrusions longer than 2 μm. Finally, two-headed spines were classified as branched. **(B)** Analysis of spines suggests their distribution per 10 μm dendrite. Control WT and c-Abl-KO neurons have significantly more mushroom than treated neurons (WT: 0.04 ± 0.01 vs. KO: 0.06 ± 0.01 spines/10 μm dendrite), slightly decreased stubby (WT: 0.17 ± 0.02 vs. KO: 0.15 ± 0.02 spines/10 μm dendrite), slightly increased thin (WT: 0.18 ± 0.03 vs. KO: 0.19 ± 0.02 spines/10 μm dendrite) and filopodium (WT: 0.04 ± 0.01 vs. KO: 0.04 ± 0.01 spines/10 μm dendrite) and slightly increased branched spines per dendrite (WT: 0.02 ± 0.01 vs. KO: 0.03 ± 0.01 spines/10 μm dendrite). After AβOs treatment, both, WT and c-Abl-KO neurons showed a significant reduction in the density of mushroom (WT: 0.03 ± 0.01 and KO: 0.03 ± 0.01 spines/10 μm dendrite) and stubby spines (WT: 0.15 ± 0.01 and KO: 0.16 ± 0.03 spines/10 μm). After AβOs treatment WT neurons presented higher density of filopodia spines than c-Abl-KO neurons (0.07 ± 0.02 vs. 0.05 ± 0.01 spines/10 μm dendrite, respectively) and maintenance of thin spines (0.17 ± 0.02 vs. 0.18 ± 0.02 spines/10 μm dendrite). Branched spines showed no changes after AβOs treatment (WT: 0.01 ± 0.002 vs. KO: 0.02 ± 0.01 spines/10 μm dendrite). **(C)** Spine type relative profile [percentage of each spine type classified in **(B)** counting 2–5 dendrites per neuron]. In WT and c-Abl-KO neurons, the population of thin spines increases (WT: 37.2 ± 2.8% *n* = 14 neurons; KO: 41.3 ± 1.5% *n* = 15 neurons; WT+AβOs: 41 ± 3.9% *n* = 13 neurons; KO+AβOs: 43.4 ± 1.7%, *n* = 18 neurons) and mushroom spines decreases after AβOs treatment (WT: 11.3 ± 2.2%; KO: 12.5 ± 1.8%; WT+AβOs: 6.6 ± 1.6%; KO+AβOs: 7.9 ± 2.3%); while filopodia spines increased (WT: 8.6 ± 2%; KO: 8.9 ± 1.4%; WT+AβOs: 13.3 ± 1.9%; KO+AβOs: 11.6 ± 1.9%); Stubby (WT: 38.1 ± 3.5%; KO: 30.9 ± 2.1%; WT+AβOs: 35.4 ± 2.5%; KO+AβOs: 32.9 ± 3.3%); and branched (WT: 4.8 ± 1.0%; KO: 6.5 ± 1.1%; WT+AβOs: 3.3 ± 0.8%; KO+AβOs: 3.9 ± 1.0%). **(D)** The graph shows spine length for mushroom, branched, thin and filopodia spines. AβOs induced longer filopodia spines (WT+AβOs: 3.22 ± 0.19 μm, KO: 3.83 ± 0.26 μm). Two-way ANOVA, ***p < 0.001. *n* = 3 independent cultures, 4–5 mice embryos per condition. non-significant: ns; *p < 0.5; **p < 0.01.

To distinguish between different types of spine morphology, we measure head, length, and neck of dendritic spines in GFP-expressing neurons (Figures 3A–D), and quantified the number of each spine type in a 10 μm dendrite section. We found a tendency for c-Abl-KO neurons to display more mushroom, branched and filopodia spines than WT neurons per 10 μm dendrite, however non-significant (Figure 3B). This trend towards increasing some spine types correlated with the augmented spine density observed before (Figure 2B). Since both, the neuron genotype and the binding of AβOs alter the number of dendritic spines, we normalized to the total population of dendritic spines within each dendrite analyzed. Then, we found that c-Abl deficiency alters the distribution of the spine population.

When we analyzed the head diameter of dendritic spines, we found that c-Abl-KO spines had wider heads compared to WT (0.46 ± 0.01 μm vs. 0.42 ± 0.01 μm in average head diameter, respectively; Supplementary Figure S4A), which correlates with an increased number of mushroom spines. Interestingly, we also found that c-Abl-KO spines were slender than WT spines, with a total length increase for longer filopodia and thin spines (Figure 3D) and an increment of the overall spine length (WT: 0.79 ± 0.04 and KO: 0.98 ± 0.05 μm spine length; Supplementary Figure S4B), probably due to the increase in thin population.

As expected, when AβOs were added to the medium, the most mature, mushroom spines were significantly affected by AβOs treatment. As the spine profile shows, mushroom decreased 42% by AβOs in WT, but also decreased 37% in c-Abl-KO neurons. Stubby spines were also significantly affected by AβOs treatment with a 7.2% less in WT neurons, while they tend to 6.5% augment in KO neurons (Figure 3C). Interestingly, the histogram of dendritic spines changed mushroom, stubby, thin and filopodia in WT vs. WT-treated neurons, while the overall pattern was similar for KO vs. KO-treated neurons. Interestingly, c-Abl deficiency increased the relative abundance of immature spines and maintained the mushroom and stubby population (Figure 3C). We observed a slight increase in the relative abundance of thin and filopodia spines in c-Abl-KO neurons when they were treated with AβOs (thin: 41% vs. 43.4%, and filopodium: 13.3% vs. 11.6% WT+AβOs vs. KO+AβOs, respectively; Figure 3C). Interestingly, the spine profile of WT-treated neurons displays a significant increase for filopodia spines (54.9% increase) as they are usually found within 10 μm dendrite (WT: 0.04 ± 0.01 vs. WT+ AβOs: 0.07 ± 0.02 spines/10 μm dendrite), in correlation with the lengthening of dendritic spines (WT: 0.79 ± 0.04 μm vs. WT+AβOs: 1.03 ± 0.07 μm length; Supplementary Figure S4B). Finally, branched spines were non-significantly affected by AβOs in both WT and c-Abl-KO neurons. These results suggest that AβOs-induced an overall decrease in the dendritic spine population of WT and c-Abl-KO neurons, but the effect becomes attenuated in the immature spine population of c-Abl-KO neurons. Whereas these immature spines, thin and filopodia together comprise 55% of the total spine population in c-Abl-KO treated neurons, they represent 54% in WT control-treated neurons. In the same line, the abundance of mature mushroom spines represents almost 7% of the overall population for WT, while 8% for c-Abl-KO dendritic spines after AβOs incubation (Figure 3C). The same as controls, spine head diameter of WT and c-Abl-KO neurons exposed to AβOs was very similar (WT+AβOs: 0.40 ± 0.02 μm and KO+AβOs: 0.37 ± 0.01 μm average spine head diameter), and no changes were evident between them. However, spine head diameter was significantly reduced in c-Abl-KO neurons exposed to AβOs compared with basal levels (Supplementary Figure S4A), in agreement with the significant reduction of mushroom spines.

Therefore, in the absence of c-Abl, treatment with AβOs enriches the immature dendritic spine population and preserves mushroom and stubby population maintaining the overall spine density, which suggests that this may be a possible mechanism for synaptic resiliency against AβOs.

### c-Abl Participates in the Reduction of Synaptic Clustering Induced by AβOs

**Figure 4:**
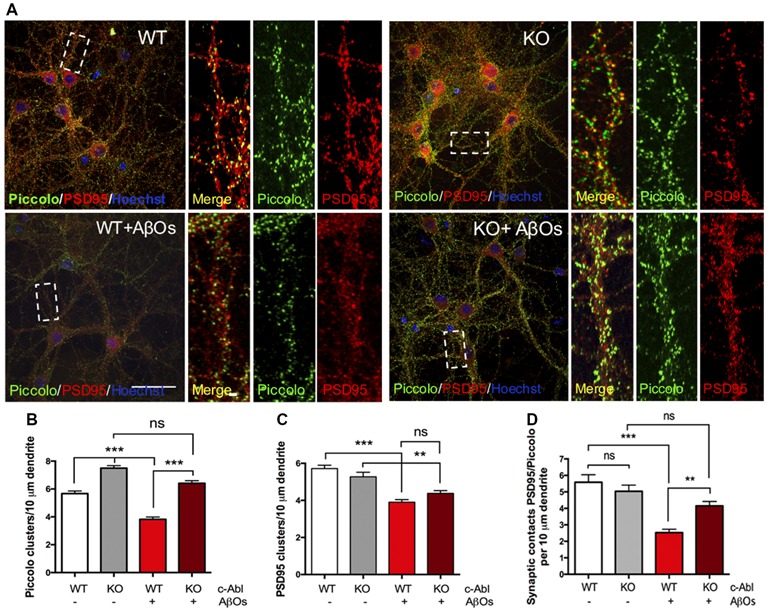
c-Abl deficiency protects Piccolo/PSD95 synaptic clustering against Aβ oligomers. **(A)** Representative example of WT and c-Abl-KO neurons stained for the pre-synaptic marker Piccolo (green) and for the post-synaptic markers PSD95 (red). **(B)** AβOs treatment does not affect the clustering of Piccolo in c-Abl-KO neurons compared with WT-treated neurons (WT: 5.67 ± 0.19 vs. WT+AβOs: 3.82 ± 0.17 clusters/10 μm dendrite, and KO: 7.45 ± 0.18 vs. KO+AβOs: 6.41 ± 0.19 clusters/10 μm dendrite; WT: *n* = 75, WT+AβOs: *n* = 81, KO: *n* = 57 and KO+AβOs: *n* = 85 dendrites; **C**). AβOs induce a significant reduction of PSD95 protein clustering in WT neurons. However, not significant reduction in the number of PSD95 clusters as observed in c-Abl-KO neurons (WT: 5.72 ± 0.18 vs. WT+AβOs: 3.91 ± 0.14 clusters/10 μm dendrite, and KO: 5.27 ± 0.24 vs. KO+AβOs: 4.38 ± 0.15 clusters/10 μm dendrite; WT: *n* = 83, WT+AβOs: *n* = 86, KO: *n* = 63 and KO+AβOs: *n* = 85 dendrites). **(D)** AβOs-induced reduction of synaptic contacts between PSD95 and Piccolo affects WT neurons (WT: 5.59 ± 0.46 vs. WT+AβOs: 2.53 ± 0.2 contacts/10 μm dendrite), while c-Abl-KO neurons maintained intact synaptic contacts (KO: 5.04 ± 0.37 vs. KO+AβOs: 4.15 ± 0.27 contacts/10 μm dendrite; WT: *n* = 23, WT+AβOs: *n* = 33, KO: *n* = 33 and KO+AβOs: *n* = 24 neurons). Two-way ANOVA and Tukey’s multiple comparison test, ***p < 0.001. Scale bar = 20 μm and 2 μm for dendrite magnifications. non-significant: ns; **p < 0.01.

In AD mice models and AD patients, AβOs induce synapse loss, decreasing levels of pre- and post-synaptic proteins and reducing the number of synaptic contacts (Sze et al., 1997; Masliah et al., 2001; Reddy et al., 2005; Calabrese et al., 2007). Therefore, we next evaluated the effect of c-Abl deficiency on AβOs synapse alteration. We examined synaptic contacts in c-Abl null hippocampal neurons after AβOs treatment by following PSD95 and Piccolo clustering (post-synaptic and pre-synaptic proteins, respectively).

c-Abl-KO neurons display increased numbers of Piccolo clusters in basal conditions in comparison to WTs. Interestingly, AβOs significantly reduced Piccolo clustering in WT neurons (3.82 ± 0.17 clusters/10 μm dendrite). While the number of Piccolo clusters in c-Abl-KO-treated neurons was very similar to its controls and significantly different from WT-treated neurons. Therefore, indicating maintenance of the pre-synaptic terminal (6.41 ± 0.19 clusters/10 μm dendrite; Figures 4A,B). Opposite results were obtained for PSD95, as c-Abl deficiency did not perturb the basal number of PSD95 synaptic clustering (WT: 5.72 ± 0.18 and KO: 5.27 ± 0.24 clusters/10 μm dendrite; Figures 4A,C). However, we observed a strong reduction in PSD95 clusters after WT neurons were treated with AβOs (WT+AβOs: 3.91 ± 0.14 clusters/10 μm dendrite) whereas, c-Abl-KO neurons treated with AβOs display less reduction in PSD95 clusters (KO+AβOs: 4.38 ± 0.15 clusters/10 μm dendrite; Figures 4A,C). However, we did not find significant differences between WT and KO-treated neurons.

On the other hand, WT neurons showed a significant reduction in PSD95/Piccolo clusters when incubated with AβOs whereas the c-Abl-KO neurons did not show synaptic loss (2.53 ± 0.2 and 4.15 ± 0.27 clusters/10 μm dendrite, respectively; Figure 4D). Colocalization analysis by Mander’s and Pearson’s correlation also showed decreased correlation for WT-treated neurons while c-Abl-KO neurons were non-significantly affected, which means protection of synaptic clustering in correlation with synaptic contact quantification (Supplementary Figures S5A,B). Our results strongly suggest that c-Abl deficiency protects the synapse.

### c-Abl Deficiency Protects Against AβOs-Induced Cell Death

**Figure 5:**
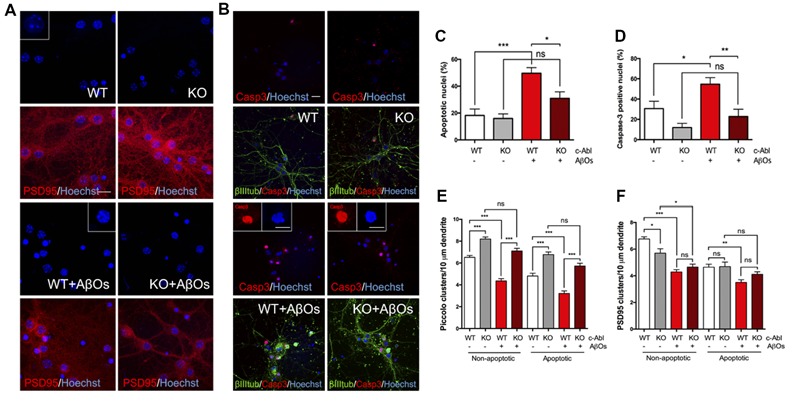
c-Abl ablation protects against Aβ oligomers-induced cell death and preserves Piccolo clusters in apoptotic and non-apoptotic neurons. **(A,B)** WT and c-Abl-KO neurons were treated with 5 μM AβOs for 5 h and Hoechst stained to label apoptotic nuclei (blue; PSD95 in red) **(A)**; and co-stained with active caspase-3 (red) antibody to confirm apoptosis; cytoskeletal protein β-tubulin is shown in green **(B)**. Graphs show the percentage of apoptotic nuclei **(C)** and the percentage of active caspase-3 nuclei **(D)** for each condition. **(E–F)** WT and c-Abl-KO neurons were classified into apoptotic vs. non-apoptotic neurons and analyzed for Piccolo **(E)** and PSD95 **(F)** cluster quantification per 10 μm dendrite. Both were significantly affected in non-apoptotic neurons, especially in WT neurons. While c-Abl-KO neurons display higher number of Piccolo clusters and were significantly preserved after AβOs treatment (WT: 6.52 ± 0.18 vs. WT+AβOs: 4.36 ± 0.21 clusters/10 μm dendrite, and KO: 8.19 ± 0.19 vs. KO+AβOs: 7.1 ± 0.24 clusters/10 μm dendrite; WT: *n* = 38, WT+AβOs: *n* = 43, KO: *n* = 29 and KO+AβOs: *n* = 42 dendrites). Apoptotic neurons displayed the same tendency for Piccolo clusters (WT: 4.81 ± 0.26 vs. WT+AβOs: 3.22 ± 0.23 clusters/10 μm dendrite, and KO: 6.77 ± 0.23 vs. KO+AβOs: 5.73 ± 0.25 clusters/10 μm dendrite; WT: *n* = 37, WT+AβOs: *n* = 38, KO: *n* = 29 and KO+AβOs: *n* = 42 dendrites). **(F)** PSD95 clusters strongly decreased in WT compared with c-Abl-KO non-apoptotic neurons while all conditions in apoptosis display significantly less clusters than healthy conditions (Non-apoptotic: WT: 6.76 ± 0.17 vs. WT+AβOs: 4.29 ± 0.17 clusters/10 μm dendrite, and KO: 5.7 ± 0.33 vs. KO+AβOs: 4.66 ± 0.22 clusters/10 μm dendrite; WT: *n* = 42, WT+AβOs: *n* = 44, KO: *n* = 36 and KO+AβOs: *n* = 42 dendrites; Apoptotic: WT: 4.65 ± 0.22 vs. WT+AβOs: 3.5 ± 0.21 clusters/10 μm dendrite, and KO: 4.69 ± 0.34 vs. KO+AβOs: 4.11 ± 0.19 clusters/10 μm dendrite; WT: *n* = 41, WT+AβOs: *n* = 42, KO: *n* = 27 and KO+AβOs: *n* = 43 dendrites; non-apoptotic: WT: *n* = 9, WT+AβOs: *n* = 10, KO: *n* = 7 and KO+AβOs: *n* = 9 neurons; apoptotic: WT: *n* = 9, WT+AβOs: *n* = 11, KO: *n* = 6 and KO+AβOs: *n* = 9 neurons). Two-way ANOVA and Tukey’s multiple comparison test, ***p < 0.001. Scale bar = 20 μm and 10 μm for magnifications. *n* = 2 independent cultures, 3–4 mice embryos per condition. ns: non-significant; *p < 0.5; **p < 0.01; ***p < 0.001.

Since AβOs-induced synaptotoxicity is linked to neuronal cell death (Yang et al., 2009), and c-Abl inhibition by Imatinib prevents AβOs-induced apoptosis (Cancino et al., 2008), we asked whether c-Abl could be responsible for apoptosis. Therefore, we quantified apoptotic nuclei using Hoechst staining (Figures 5A,C) and caspase-3 immuno-labeling (Figures 5B,D), in WT and c-Abl-KO neurons after a 5 h treatment with AβOs. As expected, in WT neurons, AβOs induced a significant increase in apoptotic nuclei (18.2 ± 4.8% control vs. 49.7 ± 4.1% AβOs-treated neurons) and in the number of caspase-3 positive cells (30.7 ± 7.2% control vs. 54.7 ± 6.5% AβOs-treated neurons). While c-Abl deficiency significantly decreased AβOs-induced apoptosis followed by apoptotic nuclei (16 ± 3.4% control vs. 31 ± 4.9% AβOs-treated neurons) and active caspase-3 positive cells (12 ± 4.1% control vs. 22.9 ± 7.1% AβOs-treated neurons; Figures 5C,D). Therefore, c-Abl deficiency protects against AβOs-induced cell death.

We also analyzed the effect of AβOs over synaptic protein removal by following PSD95 and Piccolo clusters in apoptotic and non-apoptotic WT and c-Abl-KO neurons. As before, we counted the number of clusters per 10 μm dendrite and found a strong and robust reduction of Piccolo clusters in WT neurons under AβOs, both in apoptotic and non-apoptotic neurons (Figure 5E; non-apoptotic WT: 6.52±0.18 vs. WT+AβOs: 4.36 ± 0.21 clusters/10 μm dendrite). Interestingly, the number of Piccolo clusters in c-Abl-KO neurons were maintained after AβOs treatment. Regardless of whether we examined apoptotic or non-apoptotic c-Abl-KO neurons, there was no significant difference with their controls (non-apoptotic KO: 8.19 ± 0.19 vs. KO+AβOs: 7.1 ± 0.24 clusters/10 μm dendrite). Although apoptotic neurons showed fewer Piccolo clusters than non-apoptotic neurons, after AβOs treatment all of them display a loss of Piccolo. In spite of AβOs treatment, c-Abl-KO neurons showed significantly more clusters than WT neurons in both apoptotic and non-apoptotic cells.

However, the effects of c-Abl deficiency and apoptosis on PSD95 clusters reduction induced by AβOs are less clear (Figure 5F). First, there are fewer PSD95 clusters under basal conditions in apoptotic cells compared to non-apoptotic neurons. Additionally, non-apoptotic c-Abl-KO neurons showed reduced PSD95 cluster numbers in comparison with WT under basal conditions (WT: 6.76 ± 0.17 vs. KO: 5.7 ± 0.33 clusters/10 μm dendrite). However, when treated with AβOs, both WT and c-Abl-KO non-apoptotic neurons showed fewer PSD95 clusters, with no significant differences between them (WT+AβOs: 4.29 ± 0.17 vs. KO+AβOs: 4.66 ± 0.22 clusters/10 μm dendrite). Interestingly, c-Abl-KO apoptotic neurons did not show a further reduction in PSD95 clusters in comparison with WT neurons (apoptotic KO+AβOs: 4.11 ± 0.19 clusters/10 μm dendrite).

Our results suggest that AβOs-induced Piccolo clustering reduction is significantly prevented in c-Abl-KO apoptotic and non-apoptotic neurons in comparison with WTs. On the other hand, PSD95 clusters are strongly affected by the apoptotic state of neurons derived from AβOs.

Finally, our results suggest that c-Abl mediates the signaling pathway triggered by AβOs that induces synaptic elimination and neuronal death, and could, therefore, be a relevant player in the early onset of AD.

## Discussion

Dendritic spines are specialized structures that protrude from dendrites and are the morphological correlate of excitatory synapses (Rochefort and Konnerth, 2012; Maiti et al., 2015). These structures receive and integrate information. They contain the post-synaptic machinery and signaling molecules for the propagation of signals sent out by pre-synaptic terminals and for the modulation of synaptic plasticity. Alterations in synaptic plasticity are common in neurodegenerative diseases such as the early stages of AD (Knobloch and Mansuy, 2008; Shankar et al., 2008). In AD, the decrease in synaptic density, caused mainly by oligomeric forms of the amyloid β-peptide (AβOs), shows the strongest correlation with the progression of dementia (Hardy and Selkoe, 2002; Almeida et al., 2005; Calabrese et al., 2007; Haass and Selkoe, 2007; Lacor et al., 2007; Selkoe, 2008; Ferreira and Klein, 2011; Tu et al., 2014).

As previously shown, we observed that AβOs bind to dendritic spines and induce c-Abl activation. Also, we found that *Abl1* mRNA and c-Abl protein levels increase when neurons are exposed to AβOs, just like with Aβ fibrils incubation (Figures 1D–F). Although pharmacological inhibition of c-Abl with Imatinib prevents the loss of dendritic spines induced by AβOs (Vargas et al., 2014), this inhibitor also has other targets such as Arg, c-kit, PDGFR and Src (Greuber et al., 2013; Lin and Roux, 2013). In order to dissect the contribution of c-Abl to the synaptic damage triggered by AβOs, we analyzed c-Abl null neurons. Here, we found that they exhibit increased dendritic spine density under basal conditions compared with WT neurons (Figure 2). This result is probably due to the effect that the absence of c-Abl has on the actin cytoskeleton. The C-terminal region of c-Abl directly interacts with the cytoskeleton through its G- and F-actin binding domains. c-Abl transduces several extracellular signals from tyrosine kinase receptors, promoting cytoskeleton reorganization (Jones et al., 2004; Bradley and Koleske, 2009) through the activation of GTPases like Rac1 and RhoA (Tashiro et al., 2000; Zandy et al., 2007), and the phosphorylation of the members of the WASP and WAVE2 protein families such as Abi (Takenawa and Suetsugu, 2007; Wang, 2014). Additionally, we have to consider compensatory gene effects in KO models, especially from the Arg protein (Abl1-related protein).

Results on c-Abl chemical inhibition and knockdown experiments indicate that the deficiency of this kinase protects the synapse against AβOs-damage. Indeed, c-Abl-knockdown neurons (transfected with an shRNA against c-Abl), treated with AβOs, display a slightly larger number of spines per micrometer than control neurons (Vargas et al., 2014). However, as we see in Figure 2, the dendritic spine density of c-Abl-KO and WT neurons was similarly affected by AβOs damage, even though KO neurons display more dendritic spines at basal levels. c-Abl-KO neurons were highly sensitive to AβOs, showing decreased spine density after exposure to AβOs. However, we have to consider the participation of Arg (Abl2) as a compensatory effect over the dendritic population. Therefore, AβOs-driven spine loss seems to be independent of c-Abl.

We evaluated spine morphology to explore if c-Abl deficiency exerts an effect in the presence of different types of spines driven by AβOs. As we mentioned earlier, c-Abl null neurons have more dendritic spines, including a slight increase in mushroom spines (Figures 3B,C), which are considered the most mature state of dendritic spines. This type of spine has a consolidated post-synaptic density, enriched in receptors and scaffolding proteins (Harris and Weinberg, 2012; Colgan and Yasuda, 2014). The increased presence of these proteins correlates with the enlarged spine heads present in the dendritic spines of c-Abl null neurons (Supplementary Figure S4A). Different spine shapes are a reflection of their state of maturation from the initial establishment of a dynamic synaptic contact represented by a filopodia type spine, to more mature synapses represented by mushroom spines (Harris and Kater, 1994; Fiala et al., 1998; Harris, 1999; Hayashi and Majwska, 2005; Alvarez and Sabatini, 2007). This categorization might seem rigid, but analyses of live-cell imaging show that spines are highly dynamic and may vary between thin and mushroom morphologies in timeframes that can extend from minutes to hours (Fischer et al., 1998). In response to AβOs treatment, WT neurons showed an overall decrease in the dendritic spine population. As previously described by Klein’s group, AβOs treatment induces filopodia formation (Lacor et al., 2007). We observed a significant difference for the remaining dendritic spine population to include more immature thin and filopodia spines and a shift towards these immature spines in WT neurons. Interestingly, this trend was also observed in AβOs-treated c-Abl-KO neurons. The mushroom population was highly compromised, in correspondence with overall decreased spine head widening and increased spine lengthening. However, the mushroom spine population was still higher than in WT neurons. The number of thin and filopodia spines slightly increased while stable stubby shaped spines were preserved (Figure 3B). Therefore, we hypothesize that the absence of c-Abl specifically protects the transient and highly dynamic spine population against AβOs, promoting the development of dendritic spines. This might underlie the resilience against AβOs damage showed by c-Abl-KO neurons.

However, some specific roles have been linked to c-Abl in the synapse (Vargas et al., 2018). As shown here and by others, c-Abl is located in the synapse, and its levels increase by 20 DIV neurons (Figure 1A). Using immunoelectron microscopy, Koleske and collaborators found Arg and c-Abl in the contact area of pre-synaptic terminals, mostly in dendritic spines, but not in the dendritic shaft (Moresco and Koleske, 2003). c-Abl also modulates the post-synaptic scaffold protein PSD95 through phosphorylation of Y533 (Perez de Arce et al., 2010). Therefore, we were interested in studying synapse maturation in c-Abl-KO mice when neurons are treated with AβOs. As described by Klein’s group (Lacor et al., 2007; Ferreira and Klein, 2011; Mi et al., 2017), WT neurons display a substantial reduction of PSD95 clustering when treated with AβOs while c-Abl-KO neurons display less reduction, even though the difference is non-significant between them. Interestingly, when we examined apoptotic and non-apoptotic neurons, the effect of AβOs-induced removal of PSD95 clusters was less clear. First, we found that c-Abl-KO non-apoptotic neurons show reduced PSD95 cluster numbers under basal conditions. Second, there are fewer PSD95 clusters under basal conditions in apoptotic cells compared to non-apoptotic neurons. Third, after AβOS treatment, both WT and c-Abl-KO non-apoptotic neurons show fewer PSD95 clusters. However, the differences are non-significant between these groups; this result is very similar to the one shown in Figure 4C. Interestingly, c-Abl-KO neurons showed no further decrease in PSD95 clusters compared to WT neurons after AβOs treatment. Perhaps, the absence of c-Abl prevents the removal of PSD95 associated with neuronal apoptosis, but it also leads to a decrease in basal PSD95 clustering (Perez de Arce et al., 2010), reducing the protective effect of c-Abl deficiency.

We analyzed the clusters of Piccolo, a pre-synaptic marker protein, that together with Bassoon, are scaffolding proteins of the active zone that maintain the clustering of vesicles at the nerve terminals (Gundelfinger et al., 2016). Surprisingly, c-Abl deficiency significantly contributed to the maintenance of the pre-synaptic protein Piccolo under AβOs treatment (Figure 4B). When we evaluated if the apoptotic state could affect Piccolo clustering, we found that Piccolo clusters were affected mostly by AβOs treatment in WT but not in c-Abl-KO neurons. Meaning that AβOs could induce the removal of pre-synaptic proteins like Piccolo independently of the apoptotic state of neurons and that apoptosis does not influence the protective effect promoted by c-Abl absence.

Our results on PSD95 clusters, and more clearly on Piccolo clusters, show that c-Abl deficiency prevents the loss of synapses induced by AβOs. Although the absence of c-Abl also prevents or delays apoptosis, it appears that part of the synaptic resilience seen in c-Abl-KO cells is independent of apoptosis.

In conclusion, our experiments suggest that the absence of c-Abl could stabilize Piccolo clusters while its presence could promote the removal of PSD95 clusters driven by AβOs.

We also analyzed the number of synaptic contacts (apposition between Piccolo and PSD95), and as expected, the number of synaptic contacts decreases in response to AβOs in WT neurons. However, the loss of synaptic contacts was prevented in c-Abl null neurons (Figure 4D). These results show that the reorganization of spine shapes towards immature populations induced by AβOs correlates with a smaller decrease of synaptic contacts.

We have shown here two different processes controlled in two different ways by c-Abl. One of these processes is independent of c-Abl as AβOs affect dendritic spine loss in both c-Abl-KO and WT neurons. The other process is dependent on c-Abl presence since PSD95 clustering is maintained in c-Abl-KO neurons. These processes could be differentially regulated in time and space and therefore have an opposite relation with c-Abl. We propose that the local activation of the c-Abl kinase in dendritic spines can be a determinant in the propagation of AβOs-induced damage signaling. Moreover, spine signaling and morphological changes can transduce damage signals to the rest of the cell and finally induce cell death. For this reason, we evaluated cell death induced by AβOs and found that neuronal death induced by AβOs is decreased in c-Abl null neurons (Figure 5).

Interestingly, in AD patients, synaptic dysfunction starts early on and is followed by loss of the neuronal population in cognitive regions of the brain (DeKosky and Scheff, 1990). Although it has been observed that there is a significant decrease in the number of dendritic spines in post-mortem samples of AD brains, 30–50% of older individuals have Aβ-plaques and neurofibrillary tangles but do not develop clinical dementia. Such individuals seem to have cognitive resilience that protects them against AD dementia (Mucke et al., 2000; Driscoll and Troncoso, 2011; Boros et al., 2017). Analyses of Golgi-COX stained sections of their prefrontal cortex showed that these individuals display AD pathology without the associated dementia symptoms. Even though they do not present a decrease in dendritic spine density, they do display changes in spine morphology.

Moreover, the asymptomatic women group shows an increase in the density of dendritic spines when compared with non-AD controls. Regarding the predominant spine morphology in these patients, these are mainly thin and mushroom spines, not stubby spines (Driscoll and Troncoso, 2011; Boros et al., 2017). Thus, high spine plasticity could generate synaptic resiliency against Aβ-oligomers, as we see in c-Abl null neurons in this study.

Finally, the pharmacological inhibition and genetic ablation of c-Abl kinase provide neuron resilience against damage induced by AβOs. These findings strengthen the role of c-Abl in AD and suggest that synaptic changes associated with the deficiency of c-Abl contribute to the mechanisms involved in decreasing early AD pathology and the progression of cognitive decline.

## Data Availability Statement

The datasets (generated/analyzed) for this study can be found in the Figshare repository: Raw Data c-Abl deficiency provides synaptic resiliency against Abeta oligomers.xlsx; https://figshare.com/s/7f0ddf725ca86709240d.

## Ethics Statement

All protocols were approved and followed local guidance documents generated by the *ad hoc* Chilean committee (CONICYT), and were approved by the Bioethics and Care of Laboratory Animals Committee of the Pontificia Universidad Católica de Chile (Protocol #150721002). We followed the recommendations of the Guide for Care and Use of Laboratory Animals from US Public Health Service.

## Author Contributions

DG, LV and AA designed all the experiments and DG carried out c-Abl-KO neuron experiments with and without AβOs. CF and AC-C performed western-blot and qPCR analysis. NL and DG prepared neuronal cultures of c-Abl^floxo^ mice. DG, LV and AC-C performed the formal analysis. DG, AC-C and AA participated in results, discussion and preparation of the first and final draft of the manuscript. All authors contributed to manuscript revision, read and approved the submitted version.

## Conflict of Interest

The authors declare that the research was conducted in the absence of any commercial or financial relationships that could be construed as a potential conflict of interest.
