# Supplementary material for: c-Abl Deficiency Provides Synaptic Resiliency Against Aβ-Oligomers
Source: Front Cell Neurosci. 2019 Nov 26;13:526. doi: 10.3389/fncel.2019.00526 (PMC6902026; doi:10.3389/fncel.2019.00526)
Supplement: Supplementary file 1 [file Data_Sheet_1.docx]

Supplementary Material

# Supplementary Figures and Tables

Figures, tables, and images will be published under a Creative Commons CC-BY licence and permission must be obtained for use of copyrighted material from other sources (including re-published/adapted/modified/partial figures and images from the internet). It is the responsibility of the authors to acquire the licenses, to follow any citation instructions requested by third-party rights holders, and cover any supplementary charges.

## Supplementary Figures


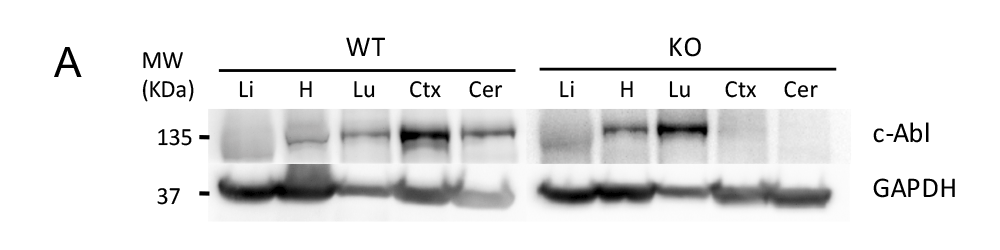


**Supplementary Figure 1. c-Abl protein expression in mice different tissues.** Homogenates from Li: liver, H: heart, Lu: lung, Ctx: brain cortex and Cer: cerebellum extracts from three months old c-Abl conditional knock-out (KO) and wild-type (WT) mice were assayed for c-Abl total protein expression.

**
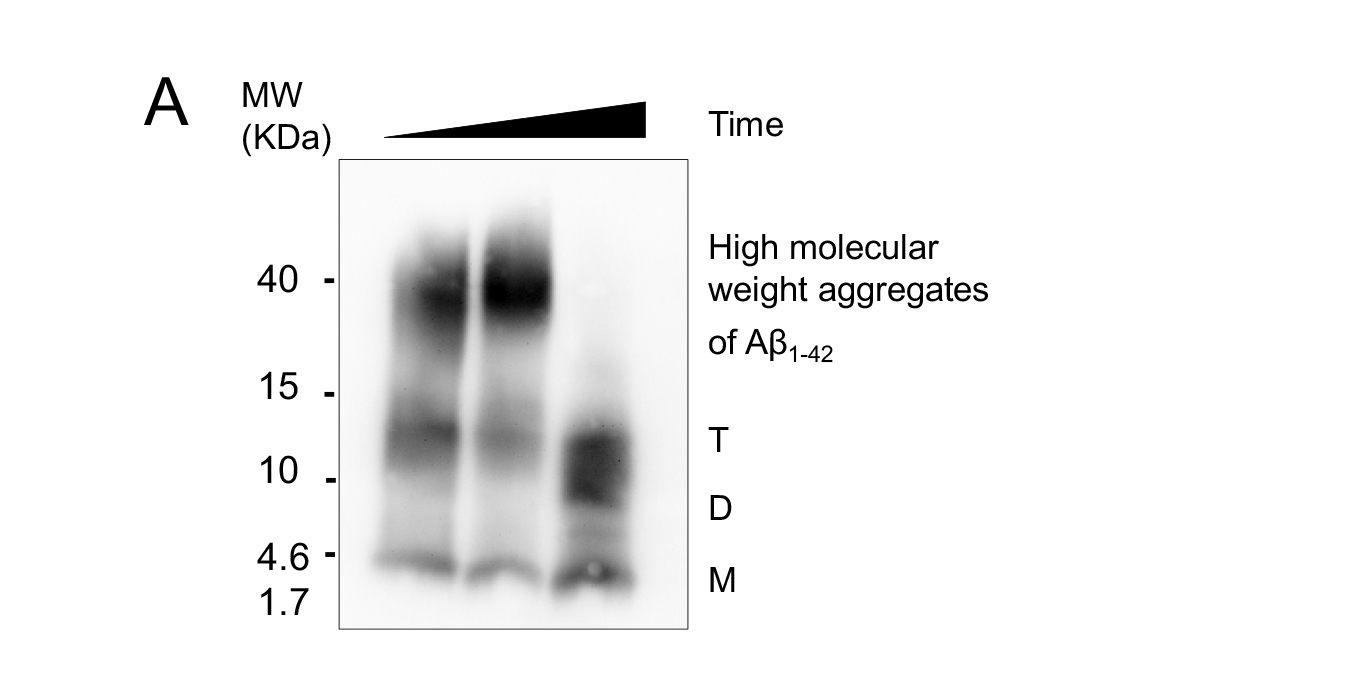
**

**Supplementary Figure 2. Preparation of Aβ_1-42_ oligomers.** Aβ_1-42_ oligomers were prepared from lyophilized human synthetic peptide and re-suspended in distilled water at 200 µM concentration (First lane). Aliquots incubated at 37°C are mostly high molecular weight aggregates and trimers of the re-suspended Aβ_1-42_ peptide (Lane 2). The aliquots incubated overnight at 4°C consist mostly of trimers and dimers, the well-known toxic oligomeric forms of the peptide (Last lane). Samples were run on denaturing Tris-tricine gels and immunoblotted with the anti-Aβ_1-42_ peptide WO2 antibody.

**
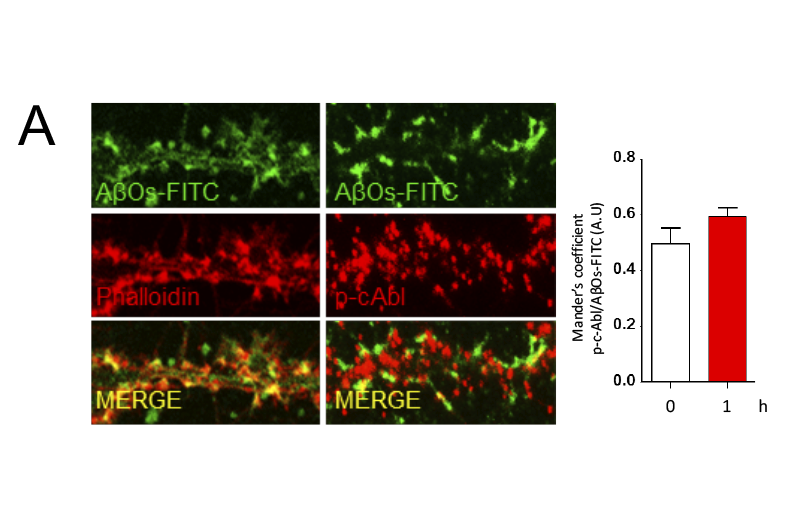
**

**Supplementary Figure 3. AβOs bound to the synapse as c-Abl activate. A.** Hippocampal 18 DIV neurons were incubated with 5 µM FITC-labelled AβOs (in green) for 1 h and immunostained for phosphorylated c-Abl at tyrosine 412, p-c-Abl and phalloidin (in red). Mander’s coefficient shows increased correlation of p-c-Abl and AβOs-FITC bound to the synapse.

**
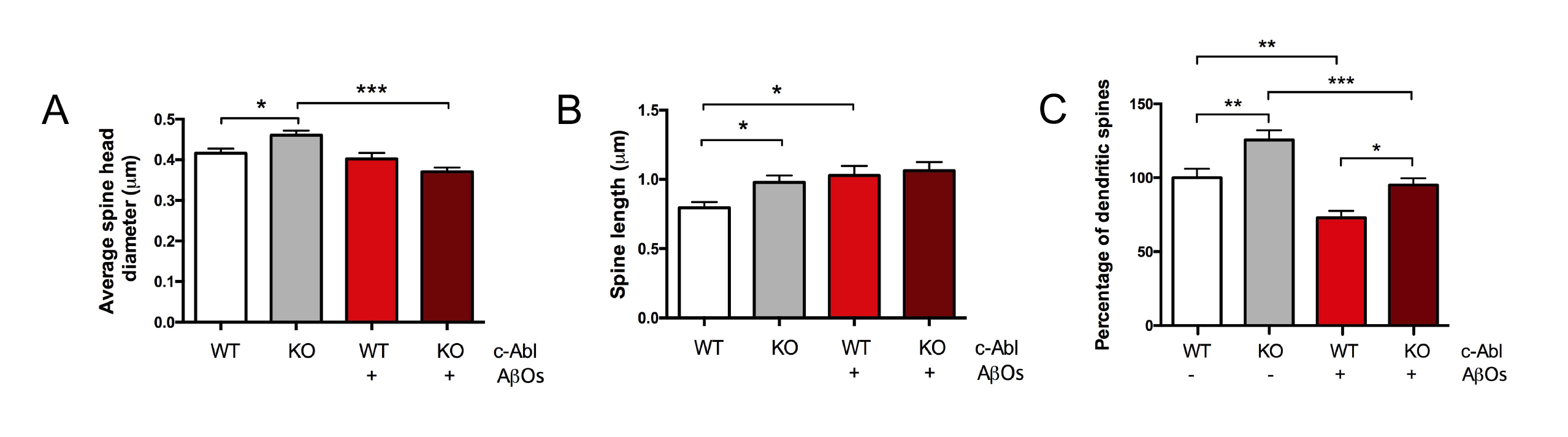
**

**Supplementary Figure 4. Spines in c-Abl null neurons are smaller in diameter but larger in length after AβOs treatment.** A, B. c-Abl-KO neurons display wider (0.46±0.01 µm, n=336 spines) (as measures by average spine head diameter) (A) and lengthier (0.98±0.05 µm) (B) spines than WT neurons (W= 0.42±0.01 µm, L= 0.79±0.04 µm, n=336 spines). AβOs-induced spine lengthening and head diameter reduction in both, c-Abl-WT (W= 0.40±0.01 µm, L= 0.98±0.07 µm, n=167 spines) and c-Abl-KO neurons (W= 0.37±0.01 µm, L= 1.06±0.06 µm, n=264 spines) One-way ANOVA. C. Percentage of dendritic spines based on n°spines/10 µm dendrite (Average percentages: WT: 100%, KO: 125.6%, WT+AβOs: 72.97%, KO+AβOs: 95.06%). Two-way ANOVA and Tukey’s post hoc *p<0.5; **p<0.01; ***p<0.001. n=3 independent cultures, 4-5 mice embryos per condition.


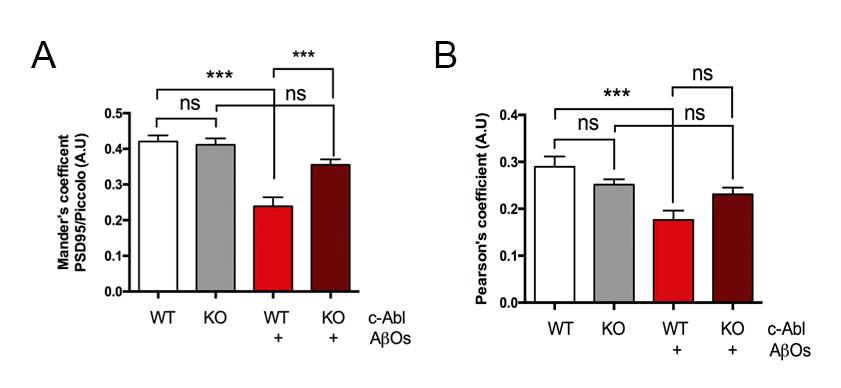


**Supplementary Figure 5. PSD95/Piccolo colocalization decreases after** **AβOs treatment and is protected in c-Abl-KO neurons.** Mander’s (A) and Pearson’s (B) coefficient for PSD95/Piccolo colocalization analysis. (WT Pearson: 0.29±0.02 Mander’s: 0.42±0.02 n=20, KO Pearson: 0.25±0.01 Mander’s: 0.41±0.02 n=30, WT+AβOs Pearson: 0.18±0.02 Mander’s: 0.24±0.02 n=20, KO+AβOs Pearson: 0.23±0.01 Mander’s: 0.36±0.02 n=29). n=2 independent cultures, 3-4 mice embryos per condition. Two-way ANOVA and Tukey’s post hoc non-significant: ns; ***p<0.001.
